# Overbenefitting, underbenefitting, and balanced: Different effort–reward profiles and their relationship with employee well-being, mental health, and job attitudes among young employees

**DOI:** 10.3389/fpsyg.2023.1020494

**Published:** 2023-03-27

**Authors:** Jie Li, Janne Kaltiainen, Jari J. Hakanen

**Affiliations:** ^1^Finnish Institute of Occupational Health, Helsinki, Finland; ^2^Faculty of Social Sciences, Tampere University, Tampere, Finland

**Keywords:** effort–reward imbalance, employee well-being, mental health, job attitudes, latent profile analysis

## Abstract

We aimed to identify different, both balanced and imbalanced, effort–reward profiles and their relations to several indicators of employee well-being (work engagement, job satisfaction, job boredom, and burnout), mental health (positive functioning, life satisfaction, anxiety, and depression symptoms), and job attitudes (organizational identification and turnover intention). We examined data drawn randomly from Finnish population (*n* = 1,357) of young adults (23–34 years of age) collected in the summer of 2021 with quantitative methods. Latent profile analysis revealed three emerging groups in the data characterized by different combinations of efforts and rewards: underbenefitting (16%, high effort/low reward), overbenefitting (34%, low effort/high reward), and balanced employees (50%, same levels of efforts and rewards). Underbenefitting employees reported poorest employee well-being and mental health, and more negative job attitudes. In general, balanced employees fared slightly better than overbenefitting employees. Balanced employees experienced higher work engagement, life satisfaction, and less depression symptoms. The findings highlight the importance of balancing work efforts with sufficient rewards so that neither outweighs the other. This study suggests that the current effort–reward model would benefit from conceptualizing the previously ignored perspective of overbenefitting state and from considering professional development as one of the essential rewards at work.

## Introduction

Steadily rising mental illness is one of the most prevalent concerns of today (GBD 2019 Mental Disorders Collaborators, 2022). For example, in Finland, mental illness is among the top reasons for applying for sickness benefits [Social Insurance Institution of Finland (SIIOF), 2020]. Furthermore, employee ill-being has increased during the COVID-19 pandemic, especially among young adults (Kaltiainen and Hakanen, 2022). Among the young, establishing a career may be challenging (Vuori and Price, 2015) and expectations towards work may be high (Twenge and Campbell, 2008). These challenges may manifest as a pressure to invest high amounts of effort in work. Thus, it is essential to understand how to promote the well-being of young adults and for this, it is necessary to acknowledge the diversity among young employees (e.g., Lyons and Kuron, 2014).

We examine heterogeneity in young adults’ experiences of efforts and rewards at work, and how such appraisals are associated with employee well-being, mental health, and job attitudes. As a theoretical framework, we draw from the model of effort–reward imbalance (ERI) which states that work stress stems from a state of failed reciprocity: the employee invests more effort into work than the rewards they receive from it (Siegrist, 1996, 2016). The ERI framework was first used to explain epidemiological outcomes but has since been adapted for (social) psychological studies of burnout (Bakker et al., 2000), work engagement (Feldt et al., 2013), job satisfaction (Ge et al., 2021), and depression symptoms (Rugulies et al., 2017). However, ERI studies have predominantly been variable-centred, focusing on the associations between ERI and other variables with the assumption that the effects are similar across participants. By using person-centred methods, we introduce the added value of unobserved population heterogeneity by identifying emerging subgroups from the population (e.g., Hofmans et al., 2020). This approach allows us to draw more detailed conclusions by examining subgroup prevalences and comparing the effects to the outcomes between the subgroups. Hence, our first contribution to the ERI literature is to examine potential subgroups from a Finnish working population of young adults who differ in their ERI states, and by doing so, help organizations and policymakers focus on issues that are associated with different combinations of efforts and rewards.

The ERI framework has focused largely on the imbalance where employees invest high amount of effort yet receive little rewards (high efforts/low rewards imbalance, “underbenefitting”; Siegrist, 2017). This focus ignores the possibility that for some, the opposite may hold true; they invest only little and receive high amount of rewards (low efforts/high rewards, “overbenefitting”). Yet, a somewhat separate stream of theorizing, the reciprocity literature, suggests that both imbalances, high effort/low reward and low effort/high reward, may lead to negative outcomes (e.g., Adams, 1965; Väänänen et al., 2005; Schaufeli, 2006). Thus, sidelining overbenefitting as a potential condition limits the understanding regarding the array of approaches to promote well-being at work. As our second contribution, we illuminate the dynamism between efforts and rewards further by also examining the potential low effort/high reward condition and thus expand the scope of the ERI model.

The ERI model proposes three reward types: esteem, job security, and promotion (Siegrist et al., 2014). We propose that these rewards do not sufficiently acknowledge the modern structure of careers, which is increasingly characterized by change of jobs and individual responsibility for one’s own career (Van der Heijden and De Vos, 2015). Accordingly, career advancement and personal development are highly valued especially among young adults (De Hauw and De Vos, 2010). As our third contribution, we postulate that especially among young adults, professional development (i.e., gaining new skills and knowledge) is a relevant reward in modern work life and will thus complement the current ERI reward structure.

In addition to the lack of studies comparing the implications of different imbalances (i.e., over-and underbenefitting) in the ERI model and literature, understanding of the implications of effort/reward (im)balances for positive mental health is also lacking. Examining both the positive and negative facets of well-being is essential, as the same psychological mechanisms may not apply similarly for both. For instance, a specific type of imbalance may not have the same (but a reversed) impact on positive and negative mental health. Examining this is necessary to achieve a more comprehensive understanding of how to promote mental health. Relatedly, no studies have examined how different ERI states can impact job boredom while studies have suggested that job boredom is especially prevalent among young employees (Harju et al., 2014). As job boredom differs from other negative facets of well-being such as burnout, we provide more nuanced insights into how different ERI (im)balances may impact employee well-being.

As our final contribution, we demonstrate the implications of belonging to different ERI profiles with three thematic sets of outcomes: (1) employee well-being (work engagement, job satisfaction, job boredom, and burnout), (2) mental health (positive functioning, life satisfaction, anxiety, and depression symptoms), and (3) job attitudes (organizational identification and turnover intentions). By doing so, we expand the explanatory capacity of the ERI model to a more holistic view of employee well-being and mental health, as well as job attitudes. To achieve these aims, we examine Finnish population data collected in the summer of 2021, which consists of young adults aged 23 to 34 (*n* = 1,357).

## Theoretical framework

The present study draws from the ERI model that views the work contract as a norm of social reciprocity (Siegrist, 1996). According to Siegrist (1996, 2016, 2017), the basic premise of the ERI model is a reciprocal relationship between employee and organization, which is characterized by an employee investing work efforts and receiving rewards from the employer. This model states that failed reciprocity as investing more efforts than receiving rewards (underbenefitting) leads to negative emotions (e.g., anger, frustration, injustice, and disappointment), stress, and adverse health effects. Work efforts include time pressure due to workload, demanding work tasks, and interruptions and disturbances at work. Rewards are divided into three categories: esteem (recognition and respect), job security (continuity of current work), and promotion (salary, promotion, and career advancement).

As acknowledged also by Siegrist (2017), the ERI model explicitly focuses on the underbenefitting aspect as failed reciprocity and ignores the overbenefitting state. The focus on underbenefitting stems from the strong reaction to loss experience, which is theorized to be more significant than reactions to overbenefitting. However, other theoretical frameworks theorize that both imbalances evoke negative reactions. For example, the equity theory, which was the basis of the ERI model, states that low cost/high gain could lead to guilt (Adams, 1965; Siegrist, 2017). Some studies in the organizational context have documented the effects of under-and overbenefitting on higher burnout (Schaufeli, 2006) and well-being (Väänänen et al., 2005). Given the importance that Siegrist (2016, 2017) places on the reciprocity principle as the basis of the ERI model, we argue that it is essential to expand the model to account for the overbenefitting state and its potential health effects beyond stress and other negative facets of well-being.

### Professional development as a reward

Rewards are viewed as part of a psychological contract with the organization (Rousseau, 1990). Sometimes referred to as an anticipatory psychological contract (De Vos et al., 2009), employees have certain reciprocity expectations of the organization. Especially younger generations may have higher expectations of such reciprocity, such as opportunities to advance their career (Twenge and Campbell, 2008; De Hauw and De Vos, 2010; Akkermans et al., 2019). Evidence suggests that employers should provide career advancement and professional development opportunities to attract younger employees (De Vos et al., 2009; De Hauw and De Vos, 2010; Zupan et al., 2022). Indeed, the ERI model includes promotional aspects as a reward, yet the emphasis is on the current employment such as salary or promotion (Siegrist et al., 2014). While career advancement in the current organization is important, we argue that gaining new skills and knowledge to strengthen one’s status in the overall labour market is a central reward for the employees.

Therefore, we argue that professional development is one of the important rewards that young adults typically seek. Notably, Siegrist and Li (2020) suggest that reward concepts in the ERI model can be open to interpretation as work contracts do not specify them in detail. Thus, professional development could be an important addition to the current ERI reward structure. We operationalize professional development as gaining new skills and knowledge to acquire expertise at work. Professional development is based on the notion of self-development, in which constantly evolving work drives employees to acknowledge gaps in their skills and to address them to the latest standard (London and Smither, 1999).

Based on the literature, we expect different subgroups of effort–reward (im)balance to emerge in the data. However, the current literature does not provide sufficient grounds to formulate a specific expectation of the number of different profiles in a working population. Such profiles may vary in the extent of the imbalance, the direction of the imbalance, and if balanced, the level of balance (e.g., equal but low/high efforts and rewards). Thus, we postulate our first research question:

*RQ1*: What kind of subgroups of effort–reward (im)balance can be identified amongst young Finnish adults?

### Employee well-being and effort–reward imbalance

Different employee well-being states are characterized by the level of activation and pleasure employees experience at work (Russel, 1980; Hakanen et al., 2018). We examine work engagement and job satisfaction as positive indicators of employee well-being. Work engagement is a high pleasure and activation state that is defined by vigor, dedication, and absorption (Schaufeli et al., 2019). Job satisfaction stems from a pleasant emotional state at work (Locke, 1969) and differs from work engagement by having lower activation. Burnout and job boredom are both low activation and unpleasant states of well-being (Hakanen et al., 2018). Schaufeli et al. (2020) define burnout as a work-related mental state that consists of four core dimensions: exhaustion, mental distance, and emotional and cognitive impairment. Job boredom is defined by daydreaming, slow passage of time, and overall feelings of boredom at work (Reijseger et al., 2013). Despite their similarity, they are empirically distinct as in comparison to burnout, job boredom is a less intense state of mind (Schaufeli and Salanova, 2014). Job boredom stems from a lack of demands and challenges whereas excessive job stressors promote burnout (Harju et al., 2022). Thus, bored employees often experience a lack of stimulation and an unpleasant state of passiveness.

Studies have shown that underbenefitting, the main hypothesis of the ERI model, is positively associated with burnout (e.g., van Vegchel et al., 2005) and negatively associated with work engagement and job satisfaction (e.g., van Vegchel et al., 2005; Kinnunen et al., 2008; Ge et al., 2021). To our knowledge, this is the first study to examine the association between ERI and job boredom. Furthermore, while previous studies have indicated that an underbenefitting state should be avoided and addressed, less is known regarding the implications of overbenefitting for employee well-being. Thus, we present our second research question:

*RQ2*: How do the emerging subgroups of effort–reward (im)balance differ in employee well-being in terms of work engagement, job satisfaction, job boredom, and burnout?

### Mental health and effort–reward imbalance

Similarly to employee well-being, also mental health includes both positive (health) and negative (illness) dimensions (Iasiello et al., 2020). Thus, the absence of one (e.g., mental illness) does not necessarily mean the presence of another (e.g., positive mental health). Whereas life satisfaction describes the affective element of mental health, positive functioning describes the psychological element (Keyes et al., 2002). Positive functioning is characterized by meaningful relationships, competence, and purposeful and meaningful life (Diener et al., 2010). Conversely, depression and anxiety represent malfunctioning (Keyes, 2007). The common attributes of depressive thoughts include the inability to feel joy, loss of meaning, and desire to escape or die (Terluin et al., 2006). The features of generalized anxiety are excessive worry and restlessness (Spitzer et al., 2006).

Some ERI studies have examined life satisfaction (e.g., Kanwal and Isha, 2022), but so far, ERI studies that examine positive mental health have been scarce. Studies of mental illness have been more common, and the evidence suggests that high (*vs* low) underbenefitting increases the risk of mental illness symptoms such as anxiety and depression (Hinz et al., 2016; Kinman, 2016; Leineweber et al., 2019). While previous studies have shown the adverse health effects of underbenefitting, for a comprehensive understanding of promoting mental health, it is important to understand how different (im)balances are associated with different indicators of both positive and negative mental health. Thus, we present our third research question:

*RQ3*: How do the emerging subgroups of effort–reward (im)balance differ in their mental health in terms of life satisfaction, positive functioning, anxiety, and depression symptoms?

### Job attitudes

Among job attitudes, turnover intention is one of the most prevalent subjects to study in the ERI research. Underbenfitting imbalance has been associated with higher turnover intentions in several studies (e.g., Kinnunen et al., 2008; Derycke et al., 2010; Leineweber et al., 2021). Yet, less is known about the implications of overbenefitting for employees’ turnover intentions. We also contribute to the nomological network of ERI by examining its association with organizational identification. Based on social identity theory, Ashforth and Mael (1989) defined organizational identification as the extent that an individual defines him-or herself in terms of membership in the organization. Put differently, organizational identification is the “fundamental binding of self-definition with the collective” (Ashforth, 2016: 362). Organizational identification is essential at work as it has been found to be associated with several outcomes (e.g., higher organizational citizenship behaviors and performance; Greco et al., 2021). Thus, we present our fourth research question:

*RQ4*: How do the emerging subgroups of effort–reward (im)balance differ in their job attitudes in terms of organizational identification and turnover intentions?

## Method

### Procedure and participants

We collected the data from the Finnish population in the summer of 2021. An invitation to participate in the study was mailed to a random sample of 12,000 young adults (aged 23 to 34) drawn from the Finnish population register. The participants were invited to participate in the study either by filling out the paper survey and mailing it back for optical reading or by responding *via* an online survey to which we provided a personal link. Altogether 1798 participants responded (15% response rate). For the present study, we included participants who were employed during the data collection period (full-time, part-time, or on-demand employment) and worked at least 10 h a week (n = 1,537). Table 1 presents sample characteristics. The data were weighted to represent the Finnish population in terms of gender, age, and residential area. This research was approved by the ethical review committee of Finnish institution of Occupational Health. In the invitation letter, we guaranteed confidentiality to the participants, emphasized that participation in the study was voluntary, and provided information about the study (e.g., only pseudonymized data is analyzed, the data is stored in secured servers, and data will be used for scientific research). We also informed that the participants have the right to inspect and verify the correctness of one’s own data, and contact details for further information.

**Table 1:** Sample characteristics (*n* = 1,357).

	%/Mean (SD)
*Age*	29.5 (3.3)
*Gender*	
Female	58
Male	42
*Education*	
Primary or secondary	35
University or doctoral	65
*Employment*	
Full-time	85
Part-time	13
On-demand	2
*Work sector*	
Public	32
Private	61
Non-profit or other	7
*Working hours per week*	36.8 (7.1)

### Measures

The items and scales for all the measures are listed in the Supplementary material. Efforts and rewards were measured using the short version of the Effort–Reward Imbalance Questionnaire (ERI-Q; Siegrist et al., 2014). The questionnaire has three items that measure work efforts (demanding qualitative and quantitative aspects of work), two items that measure esteem (recognition and respect), two items that measure job security (continuity of current work), and three items that measure promotional aspects (salary, promotion, and career advancement). We developed a two-item scale for professional development as a reward: ‘*In my work, I gain new knowledge and learn new skills*’ and ‘*In my work, I develop professionally*’.

Work Engagement was measured with three items, one depicting each dimension of work engagement (vigor, dedication, and absorption; Schaufeli et al., 2019). Job satisfaction was measured with one item, ‘*Overall, how satisfied are you with your present job?*’. Job boredom was measured by three items representing behavioural, cognitive, and affective aspects of job boredom and adapted from the Dutch Boredom Scale by Reijseger et al. (2013). Burnout was measured using the twelve-item version of the Burnout Assessment Tool (Schaufeli et al., 2020; Hadžibajramović et al., 2022). The measure includes the four core symptoms of burnout (exhaustion, mental distance, cognitive impairment, and emotional impairment) and each symptom is measured with three items.

Positive functioning was measured using the Flourishing Scale by Diener et al. (2010). This eight-item measure captures a single-factor positive socio-psychological functioning. Life satisfaction was measured by asking ‘*Overall, how satisfied are you with your life?*’. Depression symptoms were measured by six items drawn from the Four-Dimensional Symptom Questionnaire (Terluin et al., 2006). The scale captures core traits of major depression such as self-harming thoughts and a sense of worthlessness. Anxiety was measured using the seven-item Generalized Anxiety Disorder (Spitzer et al., 2006). The measure captures typical anxiety symptomology such as nervousness, anxiousness, and trouble relaxing.

Organizational identification was measured using four items drawn from Postmes et al. (2013) and Leach et al. (2008). The measure captures solidarity, satisfaction, centrality, and overall social identification with the employer organization. Turnover intention was measured with one item, ‘*I often think about resigning from my current job*’.

### Analysis

The analysis was conducted using Mplus v.8 (Muthén and Muthén, 1998/2017; RRID: SCR_015578), and maximum likelihood estimation with robust standard errors (MLR) to account for the non-normal distributions in some of the measures. We first conducted confirmatory factor analysis (CFA) to examine the factorial structure of ERI-Q with professional development as an added factor. Efforts were operationalized as a first-order factor, and rewards as a second-order factor.

To answer the research questions, latent profile analysis (LPA) was conducted to assess potential subgroups that may vary in their degree of efforts and rewards. The profile enumeration process begins with a single profile model to describe the data and more profiles are gradually added while evaluating whether the model fit improves (Spurk et al., 2020). We set 4,000 sets of random starts in the initial stage optimizations, 1,000 final stage optimizations, and 150 iterations (Hipp and Bauer, 2006).

Spurk et al. (2020) and Hofmans et al. (2020) have compiled various statistical criteria to help select the best-fitting profile solution. Lower values of Aiken Information Criteria (AIC), Bayesian Information Criteria (BIC), and Sample Adjusted Bayesian Information Criteria (saBIC) indicate a better fit, but attention should be paid to the relative decrease of these values (as opposed to selecting the model with the lowest value). The closer the entropy value is to 1, the better the separation between profiles. A statistically significant (*p* < 0.05) result in the Vuo-Lo–Mendell–Rubin likelihood ratio test (VLMR) and the adjusted Lo–Mendell–Rubin likelihood ratio test (LMR) indicates that the current profile solution (*k*) should be retained as opposed to the *k*-1 solution. Most importantly, the selected profile solution has to be congruent with theory and each added profile should have substantial meaning. The best-fitting profile solution is analysed using the automatic BCH (Bolck-Croon-Hagenaars) command which uses auxiliary variables – employee well-being, mental health, and job attitude indicators – as outcomes of the profile memberships (Asparouhov and Muthén, 2014; Bakk and Vermunt, 2015). In our study, we first standardized the means of our outcome variables and then used the automatic BCH command to estimate the means for each profile. The means are then compared across profiles by using a Wald χ2 difference test to reveal any significant differences in the outcomes between the profiles (Asparouhov and Muthén, 2014).

## Results

### Preliminary analysis

Table 2 shows the descriptive statistics, correlations, and Cronbach’s alphas. The CFA model, including efforts as a first-order construct and rewards as a second-order construct (promotion, job security, esteem, and professional development), had an acceptable fit with the data [*χ*2(df) = 201.350 (49) *p* < 0.001, root mean square error of approximation (RMSEA) = 0.048, comparative fit index (CFI) = 0.948, Tucker-Lewis index (TLI) = 0.930, standardized root mean squared residual (SRMR) = 0.062]. In this model, we estimated the covariance between the residuals of the two professional development items, as suggested by the model modification indices (Byrne, 2012).

**Table 2:** Descriptive statistics, correlations, and Cronbach’s alphas.

	*α*	*M*	SD	Scale	1.	2.	3.	4.	5.	6.	7.	8.	9.	10.	11.
1. Efforts	0.73	3.22	0.91	1–5	–										
2. Rewards	0.79	3.41	0.66	1–5	−0.19	–									
3. Work engagement	0.82	4.26	1.31	0–6	−0.13	0.50	–								
4. Job satisfaction	–	3.64	1.06	1–5	−0.23	0.56	0.54	–							
5. Job boredom	0.79	3.29	1.47	0–6	0.02	−0.34	−0.40	−0.37	–						
6. Burnout	0.86	2.21	0.56	1–5	0.39	−0.51	−0.54	−0.58	0.49	–					
7. Positive functioning	0.92	5.46	1.01	1–7	−0.07	0.38	0.36	0.36	−0.32	−0.50	–				
8. Life satisfaction	-	3.93	0.83	1–5	−0.05	0.38	0.34	0.41	−0.28	−0.44	0.74	–			
9. Anxiety symptoms	0.90	0.73	0.65	0–3	0.23	−0.32	−0.26	−0.35	0.30	0.56	−0.47	−0.48	–		
10. Depression symptoms	0.92	1.29	0.60	1–5	0.10	−0.25	−0.23	−0.30	0.25	0.39	−0.61	−0.56	0.52	–	
11. Organizational identification	0.88	3.68	0.96	1–5	−0.18	0.58	0.58	0.63	−0.45	−0.54	0.31	0.32	−0.26	−0.23	–
12. Turnover intention	–	2.40	1.27	1–5	0.33	−0.60	−0.49	−0.60	0.36	0.59	−0.30	−0.34	0.33	0.26	−0.62

*α* = Cronbach’s alpha; *M*, mean; SD, standard deviation. Correlation −0.07 is statistically significant at *p* < 0.05; correlation 0.10 is statistically significant at *p* < 0.01; correlations greater than 0.10 are statistically significant at *p* < 0.001.

### Latent profile analysis

Table 3 shows the profile enumeration process. We estimated up to seven-profile solution as solutions with more than seven profiles showed convergence issues. AIC, BIC, and saBIC did not substantially decrease after the three-profile solution (see the Supplementary material). No model had a superior entropy value, as they all ranged from 0.516 to 0.637 across solutions, indicating that the selection of the final profile solution should be based on other indices. VLMR and LMR were statistically significant in the three-profile solution, suggesting that the solution should be retained over the previous solution (two-profile), but was not significant for the four-profile solution and onwards, thus providing further support for the three-profile solution.

**Table 3:** Model fit statistics for latent profile solutions.

Profile #	LL	AIC	BIC	saBIC	Entropy	VLMR (Value of *p*)	LMR (Value of *p*)	Latent profile proportions (%)
1	−3158.729	6325.459	6346.311	6333.604	–	–	–	–
2	−3115.995	6245.991	6282.482	6260.246	0.516	0.000***	0.000***	79/21
3	−3093.677	6207.355	6259.485	6227.719	0.525	0.019*	0.024*	16/34/50
4	−3083.275	6192.550	6260.320	6219.024	0.603	0.670	0.680	2/49/16/33
5	−3077.917	6187.833	6271.242	6220.416	0.565	0.428	0.431	39/11/13/13/24
6	−3064.468	6166.935	6265.983	6205.628	0.637	0.179	0.187	1/13/14/21/34/17
7	−3060.152	6164.304	6278.991	6209.106	0.610	0.619	0.623	17/1/10/12/18/12/30

LL, Log likelihood value; AIC, Akaike information criterion; BIC, Bayesian information criterion; saBIC, Sample adjusted Bayesian information criterion; VLMR, Vuong-Lo-Mendell-Rubin likelihood ratio test; LMR, Lo-Mendell-Rubin adjested likelihood ratio test. *p < 0.05, **p < 0.01, ***p < 0.001.

**Figure 1:**
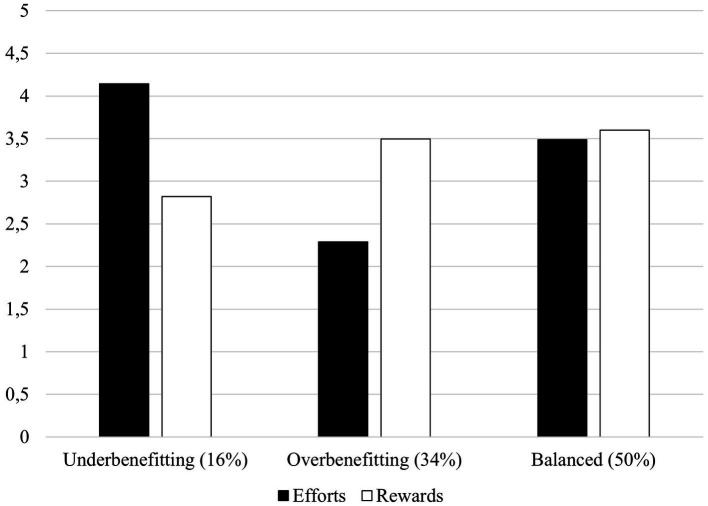
The three-profile solution for different combinations of efforts and rewards at work. Mean values are presented on the vertical axis. Percentages represent proportions from the data.

Conceptually, the three-profile solution also fits the ERI model. The solution contained the central aspects of reciprocity by including a balanced state of efforts and rewards as well as both imbalanced states. We concluded that the three-profile solution was conceptually and statistically the best fit for the data. As shown in Figure 1, the largest group in the three-profile solution represented 50% of the data and was named ‘balanced’ employees, due to the balanced state of the reported efforts and rewards. The second largest group (34%) was named ‘overbenefitting’ employees given the low efforts and high rewards in this profile. Lastly, a small group (16%) was named ‘underbenefitting’ employees due to their high efforts and low rewards.

### Profile outcomes

**Figure 2:**
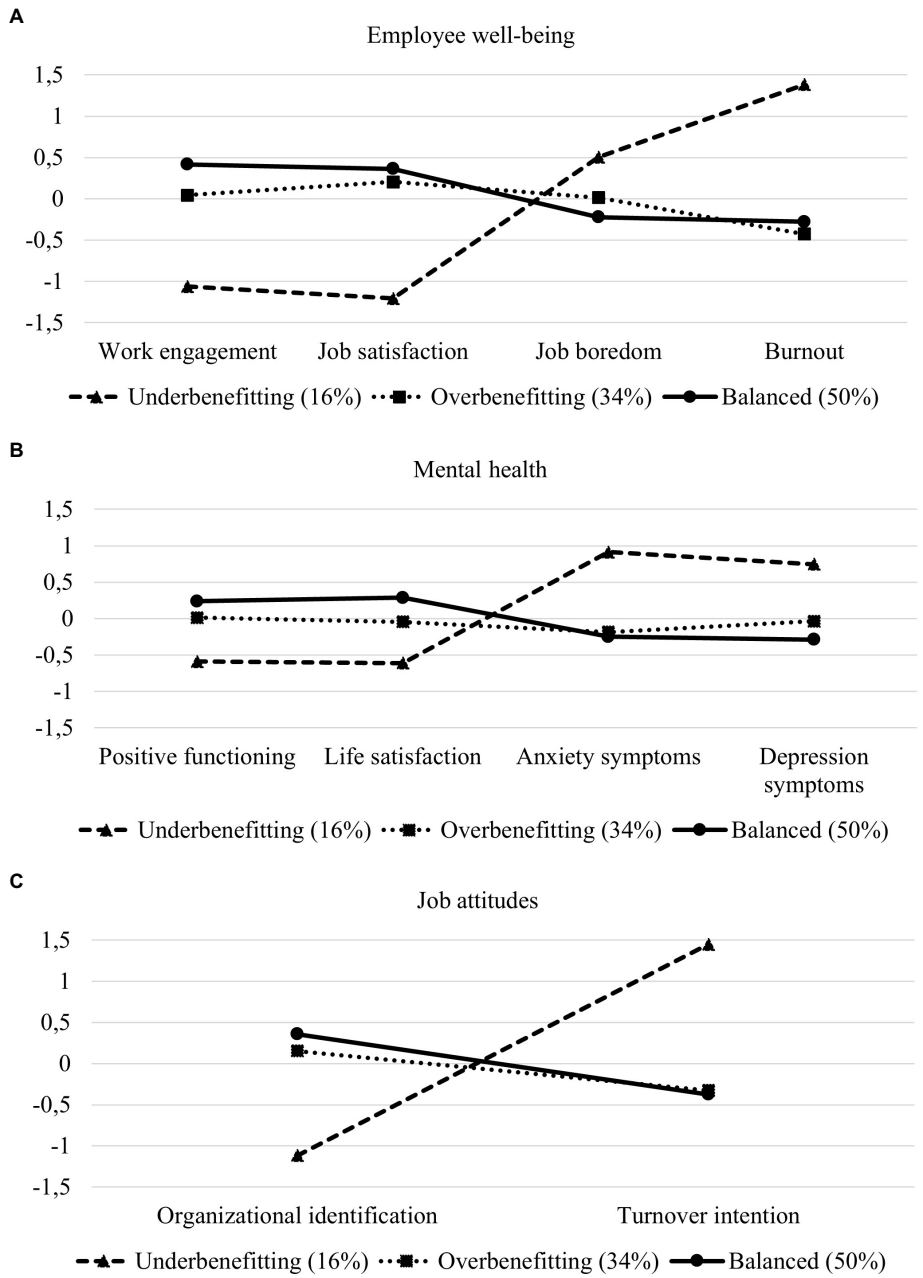
The standardized means (mean = 0, standard deviation = 1) of the outcome variables [panel **(A)** for employee well-being, panel **(B)** for mental health, and panel **(C)** for job attitudes] for each of the three profiles.

Figure 2 shows the outcomes of the three-profile solution. Outcome variables were standardized to have a mean of 0 and a standard deviation of 1. Detailed descriptions of the standardized means and standard errors can be found in the Supplementary material. Table 4 shows the results of the *χ*2 difference test. Significant differences were found in the outcome variables across the profiles.

**Table 4:** Equality tests of means across classes using automatic BCH procedure in Mplus with 2 degrees of freedom from the overall test.

	Δ*χ*2 (*p*-value)	Δ*χ*2 (*p*-value)
*Balanced*	*vs Overbenefitting*	*vs Underbenefitting*
Job satisfaction	1.576 (0.209)	108.582 (0.000)***
Work engagement	9.248 (0.002)**	74.079 (0.000)***
Job boredom	3.223 (0.073)	18.386 (0.000)***
Burnout	1.580 (0.209)	106.050 (0.000)***
Life satisfaction	6.331 (0.012)*	36.917 (0.000)***
Positive functioning	2.861 (0.091)	21.309 (0.000)***
Anxiety symptoms	0.295 (0.587)	38.955 (0.000)***
Depression symptoms	3.888 (0.049)*	19.406 (0.000)***
Organizational identification	2.676 (0.102)	90.154 (0.000)***
Turnover intention	0.166 (0.684)	176.045 (0.000)***
*Underbenefitting*	*vs Overbenefitting*	
Job satisfaction	116.110 (0.000)***	
Work engagement	57.963 (0.000)***	
Job boredom	11.872 (0.001)**	
Burnout	157.126 (0.000)***	
Life satisfaction	19.355 (0.000)***	
Positive functioning	16.638 (0.000)***	
Anxiety symptoms	49.954 (0.000)***	
Depression symptoms	15.565 (0.000)***	
Organizational identification	88.089 (0.000)***	
Turnover intention	204.026 (0.000)***	

Δ*χ*2, Chi-square difference between the two compared means. *p < 0.05, **p < 0.01, ***p < 0.001.

Employee well-being was poorest among the underbenefitting in comparison to the other two groups. They experienced the lowest work engagement and job satisfaction as well as the highest job boredom and burnout. These differences were statistically significant in comparison to the balanced and overbenefitting employees (Table 4). The balanced employees differ from overbenefitting employees by experiencing higher levels of work engagement.

Mental health was also poorest in the underbenefitting groups. They reported the lowest levels of positive functioning and life satisfaction, as well as the highest levels of anxiety and depression symptoms, as shown by the statistically significant differences (Table 4). The balanced employees differ from overbenefitting employees by experiencing higher levels of life satisfaction and lower levels of depression symptoms.

Also, the job attitudes were more negative among the underbenefitting employees. They had the lowest level of organizational identification and the highest level of turnover intentions. In terms of job attitudes, no significant differences were found between the balanced and overbenefitting employees.

## Discussion

Our study aimed to identify different effort and reward (im)balance profiles and their relationships to employee well-being, mental health, and job attitudes in a Finnish working population of young adults. Three subgroups emerged (Figure 1) with implications for employee well-being (work engagement, job satisfaction, job boredom, and burnout), mental health (positive functioning, life satisfaction, anxiety, and depression symptoms), and job attitudes (organizational identification and turnover intention; Figure 2). Balanced employees were the most prevalent profile (50%), after which the overbenefitting profile accounted for a third of the data (34%). Lastly, a smaller profile of underbenefitting employees was discovered (16%; Figure 1).

Our LPA results complement previous person-centred studies by conceptualizing overbenefitting and balanced groups as separate states, instead of examining only low or moderate underbenefitting imbalance, as in previous person-centred studies. The profiles found in this study were similar to those found in the study by Feldt et al. (2013), in which the overbenefitting profile was called ‘*Low ERI*’ due to the study’s focus on examining the risks of underbenefitting. Leineweber et al. (2019) discovered no latent profile that resembled overbenefitting. However, their largest profile included 90% of the data and was called ‘*Stable low*’, with matching effort and reward levels. Further expanding the scope of the ERI model, our three profiles had distinct outcomes, emphasizing the need for further studies as well as more theoretical consideration of the overbenefitting state in the ERI model. In addition to identifying different ERI states, we also found support that professional development would complement the ERI reward structure. Given the importance of professional development in today’s world of work (e.g., Akkermans et al., 2019), its importance for young employees’ employability, and the empirical evidence provided by our study, we suggest that professional development is a plausible addition to the ERI model.

### Is receiving excess rewards an optimal state? Comparing overbenefitting and balanced employees

Although previous ERI studies have focused heavily on the stressful state of underbenefitting and its associations with negative work-related experiences (e.g., job dissatisfaction, burnout, and turnover intention), our results indicate that also overbenefitting may not be optimal for employee well-being as it is associated with lower work engagement. Interestingly, in addition to reporting higher levels of work engagement, the balanced employees reported also considerably more investment of efforts than the overbenefitting group. Based on previous research, arguably investing less efforts than receiving rewards (i.e., overbenefit) could positively affect work engagement, as studies have shown that underbenefitting is negatively related to work engagement (e.g., Wolter et al., 2019) and as efforts correlate negatively with work engagement (as in our study). However, our findings broaden the knowledge of the dynamic relationships between ERI and work engagement by showing that receiving more rewards than investing efforts may not be optimal for work engagement either. Work engagement is a high activation state, which indicates that efforts may promote work engagement. For instance, prior studies have indicated that a sense of accomplishment and the use of one’s skills and knowledge to overcome challenges, which all necessitate investment of efforts, can be beneficial (e.g., Hakanen et al., 2021a). It could be that overbenefitting employees lack challenges and inspiring tasks which is associated with investing less effort in their work. Put differently, when an employee receives more rewards than invests effort, for promoting work engagement it would be more beneficial to provide inspiring challenges to raise the employee’s activation (e.g., LePine, 2022), rather than provide even more (excessive) rewards. Furthermore, according to the self-determination Theory (Deci and Ryan, 2000), excessive external rewards may impair intrinsic motivation. This may also explain why overbenefitting employees reported a lower motivational state, that is, work engagement.

In addition to work engagement, overbenefitting may not be optimal for mental health either. Overbenefitting was associated with lower life satisfaction and higher depression symptoms in comparison to balanced employees, suggesting differences in the affective dimensions. One explanation could be that similarly to underbenefitting, also overbenefitting leads to negative emotions such as guilt, as suggested by the equity theory (Adams, 1965). Furthermore, studies have found that prosocial behaviour (i.e., behaviour that benefits others) is beneficial for mental health (e.g., Raposa et al., 2016), suggesting that giving and feeling that one is helpful (i.e., investing efforts) promotes positive mental health. This may also explain why belonging to a profile with low efforts may not be optimal. Yet, the effects of overbenefitting on lower life satisfaction and higher depression symptoms compared to the balanced employees is relatively small, especially in comparison to the more harmful effects of underbenefitting (Figure 2). Nevertheless, future ERI studies would benefit from considering how different imbalances may affect mental health and different emotional states over time.

Overall, although our findings support the notion that underbenefitting is a more harmful state than overbenefitting, they also reveal the importance of overbenefitting. This is because a third of the sample belonged to this profile, suggesting that this state affects a large group of young employees. As for employee well-being and mental health, our results suggest that the differences between overbenefitting and balanced employees are mainly manifested in the positive aspects: more work engagement and life satisfaction in the balanced group. As the effects may vary across different types of outcomes, this finding highlights the importance to examine both negative and positive aspects rather than focus only on negative facets as most ERI studies have done thus far. Our findings demonstrate that sidelining the overbenefitting state, which characterizes a large group of employees, limits our understanding regarding the dynamics between efforts and rewards, and how they may affect well-being and mental health. However, we did not find any significant differences in organizational identification and turnover intentions between the balanced and overbenefitting employees. This suggests that for the attitudes towards the job, it is essential that the reciprocal relationship is beneficial for the individual employee, either receiving equal or more rewards than investing efforts, as both similarly promote oneness with the organization and lower intentions to abandon such a favorable relationship. In terms of the aforementioned job attitudes, underbenefitting employees had the most negative job attitudes.

### Adverse outcomes for underbenefitting employees

Our findings also provide new evidence regarding the potentially harmful implications of underbenefitting, thus expanding the nomological net of ERI research. To our knowledge, this is the first study to examine the association between ERI and job boredom. Our findings suggest that in addition to higher burnout, underbenefitting from work is also associated with higher job boredom. Job boredom and burnout share some similarities that may explain why they are in tandem. Both depict low activation and displeased states (Schaufeli and Salanova, 2014) and are similar in terms of disrupted cognitive functioning such as daydreaming in job boredom and difficulties to concentrate in burnout. Recent evidence also suggests that burnout may promote job boredom and vice versa (Harju et al., 2022) which explains why these ill-being states accommodate each other. In terms of other employee well-being dimensions, our findings also further corroborate the harmful implications of underbenefitting for work engagement, job satisfaction, and burnout (e.g., van Vegchel et al., 2005) amongst young employees.

Our study also reveals that in addition to mental illness, as examined previously (e.g., Hinz et al., 2016; Kinman, 2016), underbenefitting employees may also experience low positive mental health (positive functioning and life satisfaction). Regarding job attitudes, our findings provide new insights by suggesting that the negative consequences of underbenefitting expand to low organizational identification, in addition to previously studied higher turnover intentions (e.g., Derycke et al., 2010). Thus, the negative consequences of underbenefitting may extend beyond various health outcomes and may also concern many types of employee attitudes, such as organizational identification, that are essential not only for employees but also for the success of organizations.

### Practical implications

Previous ERI studies have recommended organizations to consider and balance employees’ efforts and rewards to avoid an imbalance of excessive efforts without sufficient rewards at work. Our results support similar conclusions with the novel insight that there may be a prevalent group of young employees (34% in our data) whose balance between efforts and rewards is not optimal due to low efforts. One key characteristic of this group may be low work engagement, which may lead to, for instance, a higher risk of unemployment and work disability (Hakanen et al., 2021b). Organizations should not only ensure that employees receive adequate rewards or use rewards as the only mean to promote balance, but also make sure that employees have the possibility and resources to invest efforts in inspiring and challenging tasks (e.g., tasks that develop skills and provide opportunities to gain accomplishments). These might make other, not-so-preferred aspects of efforts (having to deal with role conflicts, red tape, etc.) more tolerable.

Appropriate efforts with matching rewards could provide a better premise for higher work engagement, slight improvements in life satisfaction, and slightly reduced depression symptoms. However, seeking a balance by increasing employees’ efforts without considering the quality or content of such efforts is not sufficient, as employees must also feel that the reciprocal relationship is fair. Organizations should maintain an atmosphere of trust, transparency, and fairness to lower employees’ threshold to come forward if they experience low efforts or a lack of challenges in their current tasks. However, especially those who seek balance by increasing efforts should pay attention to high-quality communication (e.g., addressing personal concerns and disclosing the reasons for work-related concerns; Bordia et al., 2004).

Finally, organizations should actively seek out employees who invest more than they receive. Our results indicate that these employees are a high-risk group for employee ill-being, mental illness, and negative job attitudes. Given the findings of our study and the previous ERI literature (e.g., van Vegchel et al., 2005), a balance between efforts and rewards is important for well-being both at work and outside it.

### Limitations and future research

First, we used self-report which may have increased the risk of common method bias (Podsakoff et al., 2003). To mitigate this issue, we informed the participants that there were no right or wrong answers to reduce apprehension regarding the evaluation (less likely to respond with desirable answers). We also used measures that have demonstrated good psychometric properties, reducing the possibility that the variations in the answers were caused by instruments. In addition, the ERI-Q measure included reverse-coded items to promote more cognitive processing while answering the questionnaire and we ensured the factorial validity of the ERI-Q with the added factor of professional development by conducting a CFA. Finally, we conducted Harman’s single-factor test (Fuller et al., 2016) to examine potential common method variance. The results from exploratory factor analysis revealed that a single factor accounted for 29% of the variance, suggesting that common method variance did not substantially affect the conclusions of our study. Future studies could benefit from including objective health measures. However, such measures were not plausible in the current study, given that we collected a random postal sample from the population. Furthermore, for employee well-being and job attitudes as affective-motivational states, non-subjective measures are not available.

Second, our data were cross-sectional, which limits causal inferences. Rather, our aim was to provide a premise to further expand the ERI model. The ERI model could greatly benefit from more longitudinal person-centred studies to identify different imbalance states, how they evolve over time, their effects, and their determinants. Also from a practical point of view, investigating the consequences of different profiles and possible changes within profiles longitudinally could provide more robust information to organizations regarding the best ways to achieve a balanced state and what would be the most important benefits for balancing efforts and rewards.

Third, none of our profile solutions achieved the entropy value of 0.08, which some authors have suggested to be ideal (e.g., Ferguson et al., 2020). Thus, there was some degree of uncertainty present when separating the participants into different profiles. However, other model indicators, that is AIC, BIC, saBIC, VLMR, LMR, and profile sizes supported the solution of three profiles (e.g., Spurk et al., 2020). Furthermore, Monte Carlo simulations show that entropy is less reliable as an indicator of the most suitable profile solution compared to other model fit indices (Tein et al., 2013). While there are some uncertainties regarding the profile membership for some of the participants in our data, given the strong support indicated by several other indicators of model fit and the theory, we see that the non-optimal entropy value does not substantially threaten the validity of our findings.

Finally, our study focused on young adults (aged 23 to 34) and thus our findings may not similarly generalize to older employees. Future studies could benefit from collecting samples from the whole working-age population and also from focusing on specific groups, such as certain occupations, to further illuminate the generalizability of our findings and the dynamism within the ERI model. We suggest future studies to consider also the overbenefitting perspective, rather than focusing only on the extent of underbenefitting, as our study indicates that overbenefitting employees are distinct in their lack of positive aspects in comparison to balanced employees. Relatedly, our findings also emphasize the importance to examine positive outcomes (e.g., flourishing) in ERI studies rather than focusing solely on stress or similar negative outcomes.

## Concluding remarks

Our study provides novel insights into the dynamism between efforts and rewards at work especially by illuminating the role of overbenefitting state. Even though overbenefitting does not seem to lead to severe ill-being or negative job attitudes, it is not necessarily an optimal condition for work engagement, life satisfaction, and depression symptoms. We also found that the overbenefitting state was relatively common, as it accounted for a third of the respondents. These findings highlight the importance of expanding the ERI model further to account for different types of (im)balances. Finally, our results supported the ERI model’s previously established implications of underbenefitting imbalance and provided new insights as underbenefitting may also lead to job boredom and lower organizational identification. Furthermore, our study suggests that including professional development as an additional reward can be a valuable update of the conceptualization of the original ERI model.

## Data availability statement

The raw data supporting the conclusions of this article will be made available by the authors, without undue reservation.

## Ethics statement

The studies involving human participants were reviewed and approved by The ethical committee of Finnish Institution of Occupational Health. The patients/participants provided their written informed consent to participate in this study.

## Author contributions

All authors listed have made a substantial, direct, and intellectual contribution to the work and approved it for publication.

## Funding

This research was funded by the Finnish Institution of Occupational Health.

## Conflict of interest

The authors declare that the research was conducted in the absence of any commercial or financial relationships that could be construed as a potential conflict of interest.

## Publisher’s note

All claims expressed in this article are solely those of the authors and do not necessarily represent those of their affiliated organizations, or those of the publisher, the editors and the reviewers. Any product that may be evaluated in this article, or claim that may be made by its manufacturer, is not guaranteed or endorsed by the publisher.

## Supplementary material

The Supplementary material for this article can be found online at: https://www.frontiersin.org/articles/10.3389/fpsyg.2023.1020494/full#supplementary-material
