# Supplementary material for: Overbenefitting, underbenefitting, and balanced: Different effort–reward profiles and their relationship with employee well-being, mental health, and job attitudes among young employees
Source: Front Psychol. 2023 Mar 27;14:1020494. doi: 10.3389/fpsyg.2023.1020494 (PMC10083407; doi:10.3389/fpsyg.2023.1020494)
Supplement: Supplementary file 1 [file Data_Sheet_1.pdf]

Supplementary Material

1 Measures

**Effort-Reward Imbalance** (ERI-Q; Siegrist et al., 2014)<br>**Scale:** 1 = Strongly disagree, 2 = Disagree, 3 = Neither agree nor disagree, 4 = Agree, 5 = Strongly agree
I have constant time pressure due to a heavy work load.
I have many interruptions and disturbances while performing my job.
Over the past few years, my job has become more and more demanding.
I receive the respect I deserve from my superior or a respective relevant person.
My job promotion prospects are poor. (Reverse item)
I have experienced or I expect to experience an undesirable change in my work situation. (Reverse item)
My job security is poor. (Reverse item)
Considering all my efforts and achievements, I receive the respect and prestige I deserve at work.
Considering all my efforts and achievements, my job promotion prospects are adequate.
Considering all my efforts and achievements, my salary / income is adequate.
In my work, I gain new knowledge and learn new skills*
In my work, I develop professionally*

Note. * = self-developed items regarding professional development.

**Work engagement** (UWES-3; Schaufeli et al., 2019)<br>**Scale:** 0 = Never, 1 = few times in a year, 3 = once a month, 4 = few times in a month, 5 = once a week, 6 = daily
At my work, I feel bursting with energy
I am enthusiastic about my job
I am immersed in my work

**Job satisfaction**<br>**Scale:** 1 = Strongly dissatisfied, 2 = dissatisfied, 3 = neither satisfied nor dissatisfied, 4 = Satisfied, 5 = Strongly satisfied
Overall, how satisfied are you with your present job

**Job boredom** (DUBS; Reijseger et al., 2013)<br>**Scale:** 0 = Never, 1 = few times in a year, 3 = once a month, 4 = few times in a month, 5 = once a week, 6 = daily
At work, time goes by very slowly
I feel bored at my job
During work time I daydream

**Burnout** (BAT-12; Schaufeli et al., 2020; Hadzibajramovic et al., 2022)<br>**Scale:** 1 = Never, 2 = Rarely, 3 = Sometimes, 4 = Often, 5 = Always
At work, I feel mentally exhausted
After a day at work, I find it hard to recover my energy
At work, I feel physically exhausted
I struggle to find any enthusiasm for my work
I feel a strong aversion towards my job
I’m cynical about what my work means to others
When I’m working, I have trouble concentrating
At work, I have trouble staying focused
I make mistakes in my work because I have my mind on other things
At work, I feel unable to control my emotions
I do not recognize myself in the way I react emotionally at work
At work I may overreact unintentionally

**Positive functioning** (The Flourishing Scale; Diener et al., 2010)<br>**Scale:** 1 = Fully disagree, 2 = Disagree, 3 = Slightly disagree, 4 = Neither agree nor disagree, 5 = Slightly agree, 6 = Agree, 7 = Fully agree
I lead a purposeful and meaningful life
My social relationships are supportive and rewarding
I am engaged and interested in my daily activities
I actively contribute to the happiness and well-being of others
I am competent and capable in the activities that are important to me
I am a good person and live a good life
I am optimistic about my future
People respect me

**Life satisfaction**<br>**Scale:** 1 = Strongly dissatisfied, 2 = dissatisfied, 3 = neither satisfied nor dissatisfied, 4 = Satisfied, 5 = Strongly satisfied
Overall, how satisfied are you with your life

**Anxiety symptoms** (GAD-7; Spitzer et al., 2006)<br>**Scale:** 0 = Not at all, 1 = Several days, 2 = More than half the days, 3 = Nearly every day<br>**Instruction:** For each statement, rate how often you have experienced the following feelings during the past two weeks.
Feeling nervous, anxious or on edge
Not being able to stop or control worrying
Worrying too much about different things
Trouble relaxing
Being so restless that it is hard to sit still
Becoming easily annoyed or irritable
Feeling afraid as if something awful might happen

**Depression symptoms** (4DSQ; Terluin et al., 2006)<br>**Scale:** 1 = No, 2 = Sometimes, 3 = Regularly, 4 = Often, 5 = Very often or constantly<br>**Instruction:** During the past week, did you feel…
…that everything is meaningless
…that life is not worth while
…that you would be better off if you were dead
…that you can't enjoy anything anymore
…that there is no escape from your situation
…did you ever think "If only I was dead"

**Organizational identification** (Leach et al., 2008; Postmes et al., 2013)<br>**Scale:** 1 = Fully disagree, 2 = disagree, 3 = Neither agree nor disagree, 4 = agree, 5 = Fully Agree
I feel solidarity with my work place
It is pleasant to be an employee in my work place
Being a part of my work place is an important part of how I see myself
I identify with my work place

**Turnover intention**<br>**Scale:** 1 = Fully disagree, 2 = disagree, 3 = Neither agree nor disagree, 4 = agree, 5 = Fully Agree
I often think about resigning from my current job

References.

Diener, E., Wirtz, D., Tov, W., Kim-Prieto, C., Choi, D. W., Oishi, S., & Biswas-Diener, R. (2010). New well-being measures: Short scales to assess flourishing and positive and negative feelings. *Social indicators research, 97*(2), 143-156. doi:https://doi.org/10.1007/s11205-009-9493-y

Hadzibajramovic, E., Schaufeli, W., & De Witte, H. (2022). Shortening of the Burnout Assessment

Tool (BAT)-from 23 to 12 items using content and Rasch analysis. *BMC Public Health, 22*(1), 560. doi:10.1186/s12889-022-12946-y

Leach, C. W., van Zomeren, M., Zebel, S., Vliek, M. L., Pennekamp, S. F., Doosje, B., . . . Spears, R. (2008). Group-level self-definition and self-investment: a hierarchical (multicomponent) model of in-group identification. *J Pers Soc Psychol, 95*(1), 144-165. doi:10.1037/0022-3514.95.1.144

Postmes, T., Haslam, S. A., & Jans, L. (2013). A single-item measure of social identification: reliability, validity, and utility. *Br J Soc Psychol, 52*(4), 597-617. doi:10.1111/bjso.12006

Reijseger, G., Schaufeli, W. B., Peeters, M. C., Taris, T. W., van Beek, I., & Ouweneel, E. (2013). Watching the paint dry at work: psychometric examination of the Dutch Boredom Scale. *Anxiety Stress Coping, 26*(5), 508-525. doi:10.1080/10615806.2012.720676

Schaufeli, W. B., Desart, S., & De Witte, H. (2020). Burnout Assessment Tool (BAT)-Development, Validity, and Reliability. Int J Environ Res Public Health, 17(24). doi:10.3390/ijerph17249495

Schaufeli, W. B., Shimazu, A., Hakanen, J., Salanova, M., & De Witte, H. (2019). An Ultra-Short Measure for Work Engagement. *European Journal of Psychological Assessment, 35*(4), 577-591. doi:10.1027/1015-5759/a000430

Siegrist, J., Li, J., & Montano, D. (2014). Psychometric properties of the effort-reward imbalance questionnaire. *Germany: Duesseldorf University.*

Spitzer, R. L., Kroenke, K., & Williams, J. B. (2006). A brief measure for assessing generalized anxiety disorder: the GAD-7. *Archives of internal medicine, 166*(10), 1092-1097. doi:doi:10.1001/archinte.166.10.1092

Terluin, B., van Marwijk, H. W., Ader, H. J., de Vet, H. C., Penninx, B. W., Hermens, M. L., . . . Stalman, W. A. (2006). The Four-Dimensional Symptom Questionnaire (4DSQ): a validation study of a multidimensional self-report questionnaire to assess distress, depression, anxiety and somatization. *BMC Psychiatry, 6*, 34. doi:10.1186/1471-244X-6-34

2 **Supplementary Figure 1. Distribution of information criteria**

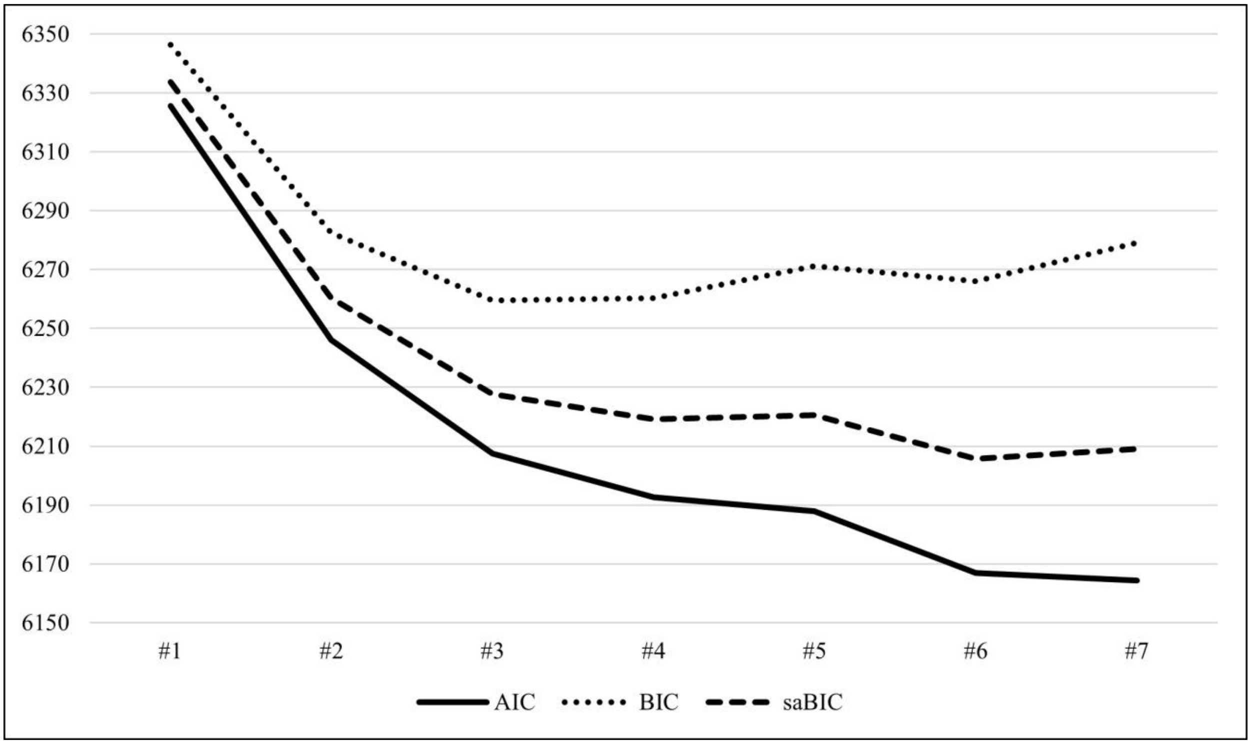

3 **Supplementary Table 1. Standardized means and standard deviations across profiles.**

	**Balanced**		**Overbenefitting**		**Underbenefitting**	
	M	SE	M	SE	M	SE
Job satisfaction	0.363	0.078	0.204	0.077	-1.206	0.112
Work engagement	0.416	0.076	0.044	0.075	-1.063	0.132
Job boredom	-0.223	0.076	0.014	0.085	0.504	0.123
Burnout	-0.278	0.070	-0.427	0.074	1.383	0.128
Life satisfaction	0.287	0.073	-0.046	0.088	-0.610	0.102
Positive functioning	0.237	0.078	0.014	0.083	-0.590	0.131
Anxiety symptoms	-0.250	0.073	-0.185	0.073	0.913	0.144
Depression symptoms	-0.288	0.072	-0.035	0.086	0.746	0.187
Organizational identification	0.359	0.074	0.154	0.079	-1.119	0.117
Turnover intention	-0.374	0.073	-0.327	0.068	1.450	0.108
